# Comprehensive Metabolomics Analysis of Xueshuan Xinmaining Tablet in Blood Stasis Model Rats Using UPLC-Q/TOF-MS

**DOI:** 10.3390/molecules23071650

**Published:** 2018-07-06

**Authors:** Jing Tan, Cuizhu Wang, Hailin Zhu, Baisong Zhou, Lingxin Xiong, Fang Wang, Pingya Li, Jinping Liu

**Affiliations:** 1 School of Pharmaceutical Sciences, Jilin University, Fujin Road 1266, Changchun 130021, China; tanjing17@mails.jlu.edu.cn (J.T.); wangcz15@mails.jlu.edu.cn (C.W.); 13578965875@163.com (H.Z.); zhoubs16@mails.jlu.edu.cn (B.Z.), xionglx14@mails.jlu.edu.cn (L.X.); 2 Department of Pathogenic Biology, Basic Medical College, Jilin University, Xinmin Street 126, Changchun 130021, China; wf@jlu.edu.cn

**Keywords:** blood stasis syndrome, Xueshuan Xinmaining tablet, UPLC-Q/TOF-MS, rats, metabolomics

## Abstract

Blood stasis syndrome (BSS) is one of the most common Chinese medicine patterns in coronary heart disease. Our previous work proved that Xueshuan Xinmaining Tablet (XXT) could treat blood stasis through regulating the expression of F13a1, Car1 and Tbxa2r. In the current study, the effect and mechanism of XXT on BSS was comprehensively and holistically investigated based on a metabolomics approach. Urine and plasma samples of 10 BBS rats treated with XXT (XT), 9 BSS model rats (BM) and 11 normal control (NC) rats were collected and then determined by UPLC-Q/TOP-MS. Multivariate analyses were applied to distinguish differentiate urinary and plasma metabolite patterns between three groups. Results showed that a clear separation of three groups was achieved. XT group was located between BM group and NC group, and showing a tendency of recovering to NC group, which was consistent with the results of hemorheological studies. Some significantly changed metabolites like cortexolone, 3α,21-dihydroxy-5β-pregnane-11,20-dione and 19*S*-hete and leukotriene A4, chiefly involved in steroid hormone biosynthesis, arachidonic acid metabolism and lipid metabolism, were found and identified to explain the mechanism. These potential markers and their corresponding pathways will help explain the mechanism of BSS and XXT treatment. This work also proves that metabolomics is effective in traditional Chinese medicinal research.

## 1. Introduction

Blood stasis syndrome (BSS), a slowing or pooling of blood [1], is one of the most common Chinese medicine patterns in coronary heart disease [2]. It plays a significant role in the development of multiple disorders and diseases [3,4]. Currently, hemorheology is an important evaluation index to estimate blood stasis [5,6]. BSS is associated with platelet-activating factor receptor genes, or with partially disturbing some metabolic pathways, etc. [2,7].

Xueshuan Xinmaining Tablet (XXT) formula, widely used in the treatment of cardiovascular and cerebrovascular diseases in China, is composed of ten traditional Chinese medicines (TCMs) including Chuanxiong Rhizoma, Salviae Miltiorrhizae Radix et Rhizoma, Hirudo, Bovis Calculus, Moschus, Pubescent Holly Root, Sophorae Flos, Total ginsenoside of ginseng stems and leaves, Borneolum syntheticum and Bufonis venenum. In our previous studies, XXT was reported to significantly ameliorate the syndrome and exhibit protective activities via decreasing hemorheological parameters in ice water-epinephrine treated rats [8]. Besides, we demonstrated that XXT had significant potent cellular protective activities against oxidative stress in human umblilical vein endothelia cells [9].

As an important platform of system biology to discover metabolic pathways associated with disease process and efficacy of drugs [10], metabolomics is applied to investigate therapeutic effects and altered potential markers in a cell, tissue or organism at a specific time or under specific circumstances [11]. It has become a pillar of the potential bridge between TCM and Western medicine, and explains the theoretical meaning of evidence-based Chinese medicine [12]. As a systemic approach, a “top-down” strategy is adopted in metabolomics to reflect the function of organisms from terminal symptoms of metabolic network and understand the complete system metabolic changes caused by interventions in holistic context, which is consistent with the holistic context of TCM [12]. Samples containing a significant amount of metabolites are always accessible biological fluids such as urine, serum or plasma [13].

In order to deeply understand the molecular mechanism of XXT against BSS, the investigation of whole biochemical variation based on metabolomic approach was comprehensively and holistically conducted in the present study. The traditional ice bath-epinephrine method was used again to make acute BSS model in rats, and the hemorheological parameters were measured to assess the success of modeling. Urine and plasma metabolic profile was assessed by using ultra performance liquid chromatography-quadrupole time-of-flight mass spectrometry (UPLC-Q/TOF-MS). And finally, the changed urine and plasma endogenous metabolites induced by XXT treatment were identified to help explain the mechanism of XXT treatment against BSS.

## 2. Results 

### 2.1. Effects of XXT on Hemorheological Parameters in Rats with BSS

**Figure 1:**
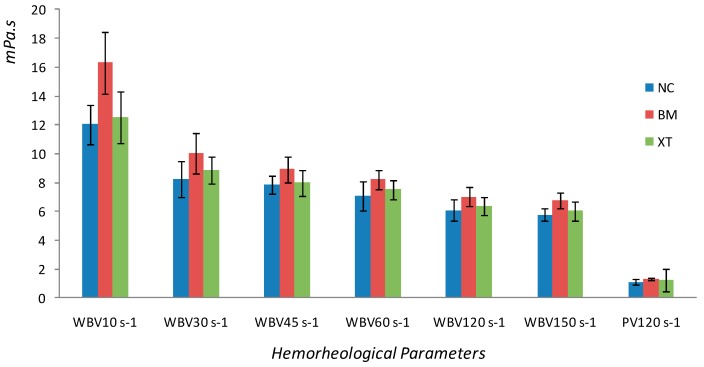
Effects of XXT on whole blood and plasma viscosity in rats with BBS.

Both whole blood viscosity at all shear rates and plasma viscosity were significantly higher in BM group than those in NC group (*P* < 0.05), and were significantly decreased in XT group compared with BM group (*P* < 0.05) (Table 1; Figure 1). Hematocrit and EAI were remarkably enhanced in BM group compared to those in NC group, and the rats pretreated with XXT demonstrated lower levels than those in BM group (*P* < 0.05) (Table 2).

### 2.2. Validation of UPLC-Q/TOF-MS

In the current study metabolomics studies were performed using UPLC-Q/TOF-MS of urine and plasma in both positive and negative mode. To evaluate the system consistency, seven species of ions were monitored as extracted ion chromatograms through the entire data set of QC injections in these two samples in both positive and negative ion modes. To cover the whole analysis process, the extracted ion chromatographic peaks of seven ions with high abundances were selected from different spectral regions. The exact mass/retention time (EMRT) pairs of these ions in urine samples were *m*/*z* 202.0482/2.11 min, *m*/*z* 539.0892/5.73 min, *m*/*z* 342.1943/8.96 min, *m*/*z* 415.2116/13.40 min, *m*/*z* 330.3372/18.39 min, *m*/*z* 554.1737/23.96 min and *m*/*z* 497.3827/27.69 min in positive ion mode, and *m*/*z* 203.0023/1.76 min, *m*/*z* 207.9947/4.87 min, *m*/*z* 485.1388/9.60 min, *m*/*z* 397.2042/12.21 min, *m*/*z* 309.1735/16.36 min, *m*/*z* 253.2162/22.54 min and *m*/*z* 283.2640/26.73 min in negative ion mode, respectively. In addition, EMRT pairs for plasma samples were *m*/*z* 309.0218/3.97 min, *m*/*z* 513.0740/5.86 min, *m*/*z* 226.1788/9.79 min, *m*/*z* 227.0918/13.43 min, *m*/*z* 515.3132/17.88 min, *m*/*z* 529.3746/23.25 min and *m*/*z* 554.3790/27.33 min in positive mode, and *m*/*z* 212.0010/2.36 min, *m*/*z* 417.1180/5.27 min, *m*/*z* 496.2726/9.40 min, *m*/*z* 352.2155/13.37 min, *m*/*z* 397.2250/17.54 min, *m*/*z* 305.2481/23.68 min and *m*/*z* 913.5844/27.90 min in negative mode, respectively, covering the whole analysis process. The relative standard deviations (RSDs) of retention times and peak areas of the seven selected ions were 0.11–2.57% and 0.38–9.21%, respectively. 

The injection precision was evaluated by analyzing five successive injections of the same QC sample. For the urine samples, the RSDs ranged from 0.36% to 2.87% for the peak intensity and from 0.02% to 0.31% for the retention time in ESI^+^ and from 0.14% to 2.98% for the peak intensity and from 0.01% to 0.35% for the retention time in ESI^−^. For the plasma samples, the RSDs of the peak areas and the retention times were 0.21~1.46% and 0.02~0.19% in ESI^+^, 0.21~2.43% and 0.02~0.51% in ESI^−^.

The reproducibility of sample preparation was estimated by detecting five parallel replicates of a urine and a plasma sample, respectively. For urine samples, the RSDs of the peak intensities were 1.65~4.12%, those of the retention time were 0.23~0.87% in ESI^+^, while were 0.87~2.36% and 0.09~0.67% in ESI^−^. While, for the plasma samples, the RSDs of the peak intensities ranged from 0.32% to 4.45% in ESI^+^ and from 0.18% to 1.69% in ESI^−^, and the RSDs of the retention times were 0.09~3.72% in ESI^+^ and 0.08~1.31% in ESI^−^.

The post-preparation stability of the sample was assessed by analyzing one sample that was left in the autosampler held at 4 °C for 0, 4, 8, 10, and 12 h. The RSDs of the peak intensity and the retention time were 1.34~4.76% and 0.09~0.45% in ESI^+^, while 0.87~5.09% and 0.08~0.42% in ESI^−^.

**Figure 2:**
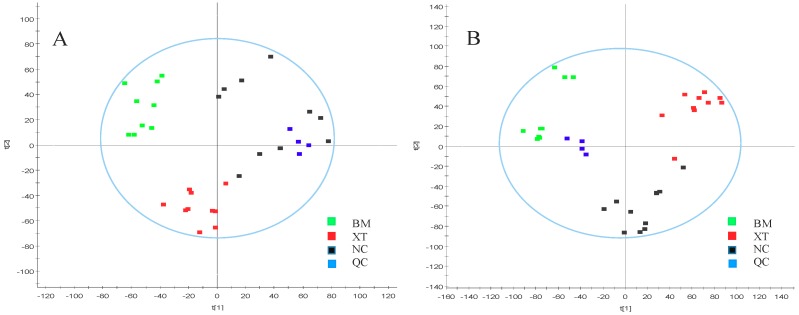
PCA score plots of urine metabolic profiling of NC, BM, XT and QC in positive mode (**A**) and negative mode (**B**).

**Figure 3:**
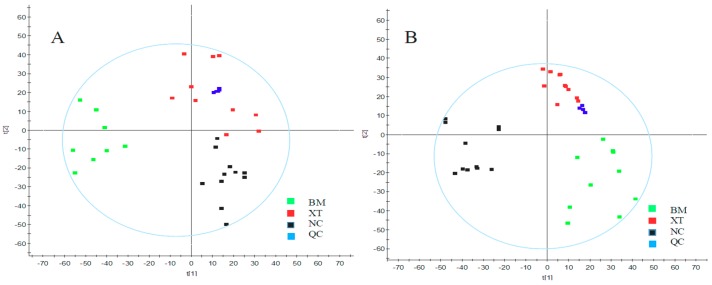
PCA score plots of plasma metabolic profiling of NC, BM, XT and QC in positive mode (**A**) and negative mode (**B**).

The above results of the method validation showed that the UPLC-Q/TOF-MS method exhibited good precision, reproducibility and stability, which could be used for the analysis of a great number of metabolomic samples. Furthermore, PCA analysis was applied to observe and give further evidence of the system stability. As shown in Figure 2 and Figure 3, the QCs were closely clustered in both positive and negative ion mode in both urinary and plasma samples. All these results were satisfactory and showed good reproducibility and stability, and QC samples also showed that analytic methods of both bio-samples could be used for large-scale metabolomics study.

### 2.3. Metabolic Profiling of UPLC-Q/TOF-MS

The current comprehensive metabolomic study was performed via UPLC-Q/TOF-MS in both positive and negative ion modes in both urine and plasma. As shown in Figure 2 and Figure 3, clear metabolite separation of these three groups could be observed via the analysis of the unsupervised method PCA in positive and negative modes in both urine and plasma samples. 

**Figure 4:**
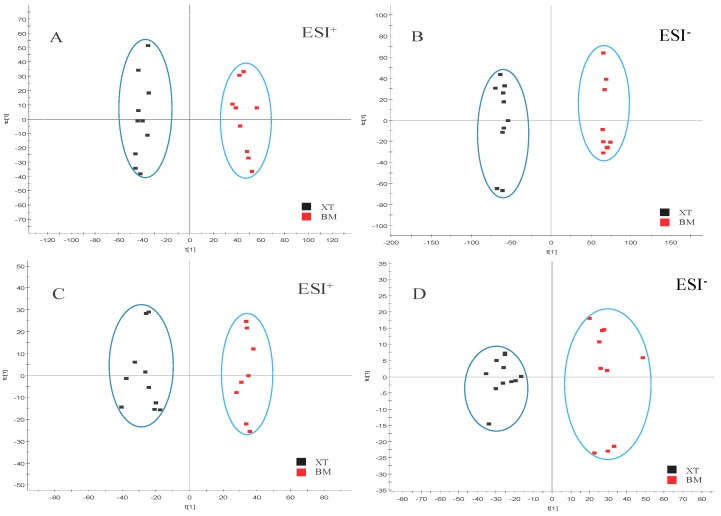
OPLS-DA score plots of urine metabolic profiling (**A**,**B**) and blood metabolic profiling (**C**,**D**) of BM and XT.

**Figure 5:**
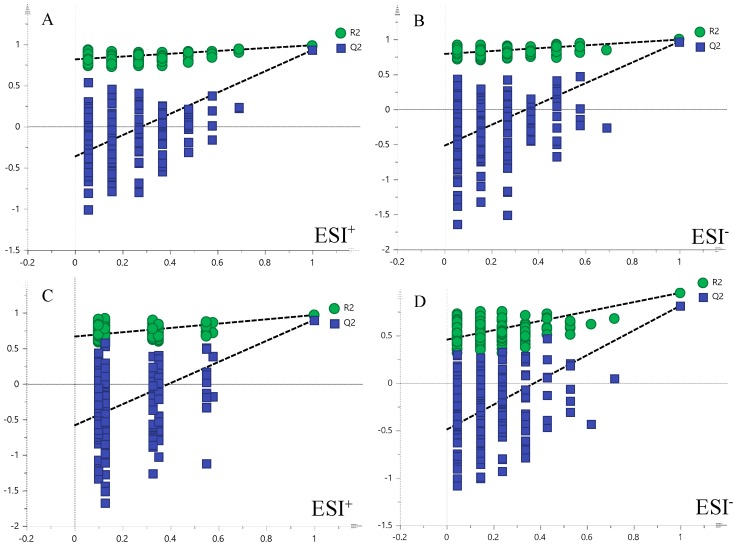
The permutations plots of the OPLS-DA models of urine (**A**,**B**) and plasma (**C**,**D**), respectively.

The supervised pattern recognition approach OPLS-DA can visualize and depict general metabolic variation between two groups. In OPLS-DA score plots, each spot represents a sample. As seen from Figure 3, the XT group was clearly separated from the BM group in both positive and negative modes in urine and plasma samples, indicating that metabolic profiles of rats that received XXT pre-treatment in XT group were significantly changed compared with those of BM group. The model parameters were R^2^Y = 0.99, Q^2^ = 0.93 in urinary positive mode (Figure 4A); R^2^Y = 1.00, Q^2^ = 0.97 in urinary negative mode (Figure 4B); R^2^Y = 0.97, Q^2^ = 0.90 in plasma positive mode (Figure 4C) and R^2^Y = 0.95, Q^2^ = 0.81 in plasma negative mode (Figure 4D), which indicated good ability of prediction and reliability of the model. The permutations plots showed all blue Q2-values to the left were lower than the original points to the right, indicating that the original models were valid (Figure 5).

### 2.4. Identification of BSS-Related Metabolites and Involved Pathway

**Figure 6:**
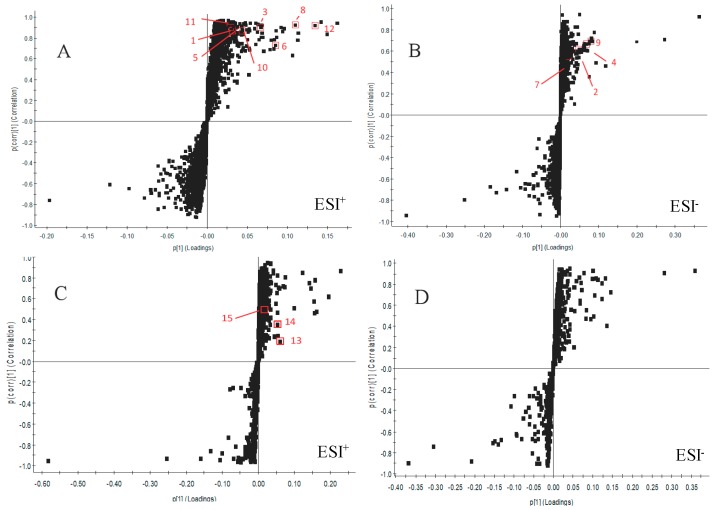
OPLS-DA *S*-plots of urine metabolic profilings (**A**,**B**) and blood metabolic profiles (**C**,**D**) of the XT group and BM group in ESI^+^ and ESI^−.^ (the numbers of potential biomarkers correspond to the list in Table 3).

To identify the metabolites contributing to the discrimination, S-plots was generated (Figure 6). Each spot in OPLS-DA score plots and S-plots represents a variance. The importance of each variance to classification is determined by the value of variable importance in the projection (VIP) and metabolites with VIP value above 1.0 and *P* value below 0.05 were considered as potential markers.

**Figure 7:**
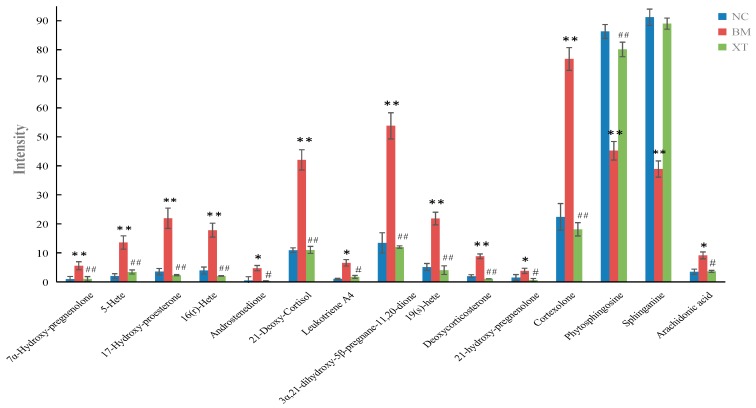
The normalized intensity of differential metabolites in different groups (* *P* < 0.05, ** *P* < 0.01 vs. NC group; ^#^ *P* < 0.05, ^##^ *P* < 0.01 vs. BM group).

The possible molecular formula of the potential markers were calculated by high-accuracy quasi-molecular ion within mass error of 10 ppm and in order to eliminate the interference fractional isotope abundance was detected by Q/TOF-MS. There were a total of 15 potential markers identified in urine and plasma. On the one hand, ten of the putative markers were confirmed by matching their retention times and accurate mass measurements with available reference standards. In the other hand, other five putative structures of these metabolites were identified by comparing accurate molecular weight and tandem mass spectrometry data obtained in this study with the information recorded in biochemical databases (HMDB). MS/MS spectrum of the potential markers were shown in Supplementary Materials. For example, the potential markers at *m*/*z* 333.2421 contained four major fragment ions at *m*/*z* 315, 287, 285 and 269. The fragment information was found to be most similar to 17-hydroxyprogesterone. The high-abundance fragment ions at *m*/*z* 315 and 269, respectively, represent [C_21_H_31_O_2_]^+^ and [C_19_H_25_O]^+^. There are another two major fragment ions at *m*/*z* 287 [C_21_H_31_O]^+^ and *m*/*z* 285 [C_21_H_29_O]^+^, further indicating that this potential markers is 17-Hydroxyprogesterone. Unfortunately none of the potential markers was identified from the blood metabolic profiling in ESI^−^ mode, so there is no legend of relevant metabolites in Figure 6D. As seen from the summary of the information of potential markers in Table 3, 7α-hydroxypregnenolone, 5-HETE (hydroxeicosatetraenoic acid), 17-hydroxyproesterone, 16(*R*)-HETE, androstenedione, 21-deoxycortisol, leukotriene A4, 3α,21-dihydroxy-5β-pregnane-11,20-dione, 19(*S*)-HETE, deoxy-corticosterone, 21-hydroxypregnenolone, cortexolone and arachidonic acid were significantly decreased in the XT group compared with those in BM group while showed no differences compared with those in NC group. On the contrary, phytosphingosine and sphinganine in the XT group were significantly increased compared with those in BM group and showed no difference compared with those in NC group. The normalized intensity of differential metabolites in different groups were shown in Figure 7.

### 2.5. Biochemical Interpretation

**Figure 8:**
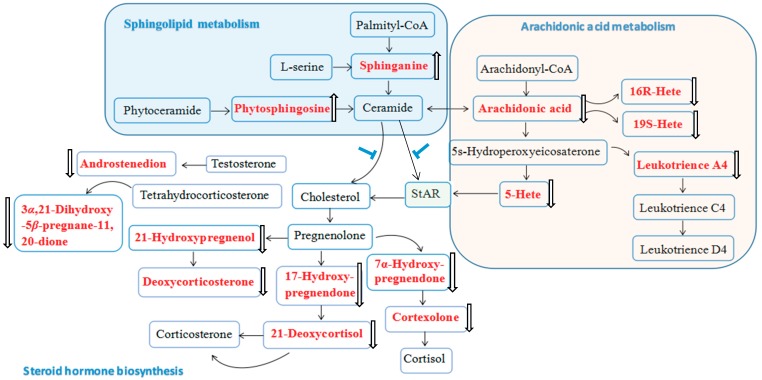
Correlation networks of potential markers in response to therapeutic effects of XXT on BSS rats according to the databases. The red marked metabolites refer to the detected potential biomarkers by UPLC-Q/TOF-MS technique (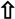: over-expressed in XT compared with BM; 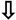: down expressed in XT compared with BM; 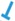: inhibiting the expression of Cholesterol/StAR).

A seen from the summary of the information of potential markers in Table 3, 7*α*-hydroxy-pregnenolone, 5-HETE, 17-hydroxyproesterone, 16(*R*)-HETE, androstenedione, 21-deoxycortisol, leukotriene A4, 3*α*,21-dihydroxy-5*β*-pregnane-11,20-dione, 19(*S*)-HETE, deoxycorticosterone, 21-hydroxypregnenolone, cortexolone and arachidonic acid were significantly decreased in the XT group compared with those in BM group while showed no difference compared with those in NC group. On the contrary, phytosphingosine and sphinganine in the XT group were significantly increased compared with those in the BM group and showed no differences compared with those in the NC group. A total of four metabolic pathways were constructed according to the web-based MetaboAnalyst method for visualizing the metabolomics results. Among the four pathways, arachidonic acid metabolism with an impact value of 0.40, steroid hormone biosynthesis with an impact value of 0.25 and sphingolipid metabolism with an impact value of 0.14 were selected as the most important metabolic pathways (Figure 8).

## 3. Discussion

According to TCM theory, BSS is mainly triggered by abnormalities of blood flow and viscosity which are initially induced by anger emotions and exogenous pathogenic factor such as a cold environment. The model in this study is established based just on this theory. The current study showed that XXT could reduce BSS-induced blood hyperviscosity and thereby decreasing the intrinsic resistance of blood flow. What’s more, a significant reduction in hematocrit in the XT group compared to the BM group was observed, indicating that the amelioration effect of XXT on WBV might be, in part, due to the hematocrit decrease. RBCs aggregation was reflected by WBV at low shear rates, and the decrease in EAI indicated that the reduction in WBV at a low shear rate in XXT-pretreated rats might be associated with the inhibition of RBCs aggregation [14]. PV is also one of the determinants of WBV, so the decline in WBV was partly related to the inhibition of a rise in PV by XXT pretreatment.

Arachidonic acid is a polyunsaturated fatty acid present in the phospholipids of cellular membranes. Under normal conditions, there is almost no free arachidonic acid [15]. When BSS occurs, the protein kinase C pathway is activated causing the activation of phospholipase A2, which results in the hydrolysis of membrane phospholipids and the release of arachidonic acid [16]. Cyclooxygenase (COX) is a key enzyme involved in arachidonic acid metabolism. When an exogenous stimulus occurs, under the catalysis of COX prostaglandin (PG) and subsequent thromboxane A2 (TXA2) are generated from arachinodic acid. PG has strong ability of dilating blood vessels and inhibiting platelet function, and TXA2 can constrict blood vessels and promote platelet aggregation [17]. In additional, leukotriene can also trigger contractions in the smooth muscles in blood vessels and increase vascular permeability [18]. In the current study, compared with the NC group, increased arachinodic acid, leukotriene A4, 5-HETE, 16(*R*)-hETE and 19(*S*)-hETE levels were observed in the BM group, which indicated that BSS caused a disturbance of arachinodic acid metabolism. At the same time, lower levels of these metabolites were found in the XT group, suggesting that XXT can normalize the interrupted pathway and may thus decrease the higher hemorheological indexes in BSS rats. Compared with the report, after the treatment of a famous herb pair Gui-Hong, all hemorheological indexes and the arachidonic acid metabolism and sphingolipid metabolism returned to normal levels [15], which was similar to our observation.

Steroidogenesis is the biological process by which steroids are generated from cholesterol. Upon reaching the outer mitochondrial membrane, the delivery of cholesterol to the inner mitochondrial membrane and the conversion of cholesterol into pregnenolone is mediated by the steroidogenic acute regulatory protein (StAR) [19]. Regardless of cellular sources, arachidonic acid is critical for steroidogenesis and StAR expression [20]. In contrast, the block of arachidonic acid release leads to the inhibition of StAR expression and steroidogenesis [21]. In our study, eight endogenous metabolites in steroid hormone biosynthesis were found to be elevated in the BM group compared to the NC group, and that these metabolites were lower after the pretreatment of XXT (*P* < 0.05). To the best of our knowledge, this is the first study revealing that BSS can induce the disturbance of steroid hormone biosynthesis in vivo and that TCM formula can ameliorate BSS via normalizing this disturbed pathway, which provides new evidence for the multi-target therapy potential of TCMs.

We also noted an interesting phenomenon whereby another two metabolites, phytosphin- gosine and sphinganine, were lower in the BM group compared to the NC group (*P* < 0.05), and returned to normal after XXT treatment. These two metabolites are involved in sphingolipid metabolism and can affect the downstream metabolite ceramide that can serve as an inhibitor of StAR expression and of the conversion of cholesterol into pregnenolone [22], which was supported by our findings.

## 4. Materials and Methods

### 4.1. Materials

XXT (Lot number: 160402) was provided by Jilin Huakang Pharmaceutical Co., Ltd (Jilin, China). Heparin sodium (Lot number: 20151224) was obtained from YM Biological Technology Co., Ltd. (Shanghai, China). Epinephrine (Lot number: 1611281) was obtained from Tianjin Pharmaceuticals Group Co., Ltd (Tianjin, China). Chloral hydrate (Lot number: 2017) was obtained from Biosharp Co., Ltd (Shenyang, China). Acetonitrile suitable for UPLC-MS were purchased from Fisher Chemical Company (Shanghai, China). Deionized water was purified using a Milli-Q water purification system (Millipore, Billerica, MA, USA).

Three standard compounds including 5-HETE (110642-201622), 16(*R*)-HETE (111675-201602), 19(*S*)-HETE (110751-201716) were purchased from Xi’an Ruixi Biological Technology Co., Ltd. (Xi’an, China). Two standard compounds including 3*α*, 21-dihydroxy-5*β*-pregnane-11,20-dione (100213-201705), and deoxycorticosterone (110823-201706) were purchased from Shanghai Zhen Zhun Biological Technology Co., Ltd. (Shanghai, China). Four standard compounds including 21-deoxy-cortisol (100120-201710), cortexolone (100534-201709), phytosphingosine (112562-201710), and sphinganine (100018-201601) were purchased from Beijing Century Aoke Biological Technology Co., Ltd. (Beijing, China). Arachidonic acid (110112-201502) was provided by Shanghai Aladdin Biochemical Technology Co., Ltd. (Shanghai, China).

### 4.2. BSS Model Construction and Drug Administration

A total of 66 male Wistar rats weighing 180–220 grams were purchased from the Animal Center of Norman Bethune Medical College of Jilin University and were habituated to their living surroundings for one week. Rats were kept under controlled conditions (22 ± 2 °C relative humidity 40–60%, 7 a.m. to 7 p.m. alternate light-dark cycles, and food and water *ad libitum*). The animal experiment was conducted according to the guide for the administration of laboratory animals (Directive 86/609/EEC on the Protection of Animals Used for Experimental and Other Scientific Purposes, 1986) and was approved by the Institutional Animal Care and Use Committee (IACUC) (ethic approval number: JL2018E2341) of Jilin University, China. The rats were randomly divided into three groups (n = 22 per group) including normal control group (NC), BSS model group (BM) and XXT (1.40 g/kg body weight, diluted in distilled water) treated group (XT). Rats in XT group were intragastrically treated with XXT aqueous solution (5 mL/kg) once daily for eight days, and rats in NC and BM groups were treated with distilled water (5 mL/kg) [8]. BSS was induced by placing rats in BM and XT group in ice-cold water for 5 min daily for eight days and keeping their heads outside water surface, and then being subcutaneously injected with epinephrine at a dose of 1 mg/kg at Day 8 [23]. Rats in NC group was only subcutaneously injected with an equal volume of saline. 

### 4.3. Sample Collection and Processing

After the last administration of epinephrine, the surviving rats in each group were respectively 22, 18 and 20. Then half of the rats of each group were anesthetized with 10% chloral hydrate (3 mL/kg) [8]. Blood for hemorheological investigation was collected from abdominal aorta and was kept in tubes containing heparin sodium dilution (20 U/mL), while blood for metabolomics analysis was collected from abdominal vein. Plasma was separated from blood by centrifugation at 3000 rpm at 4 °C for 10 min. The rest of rats in each group were used to collect urine samples. The rats were put in metabolic cages and were fasted and allowed with water *ad libitum* for 24 h. During this time, urine samples were collected into ice-cooled tubes containing 0.5 mL of 2% sodium azide [24], and were then immediately stored at −80 °C until analysis.

### 4.4. Model Assessment

Whole blood viscosity (WBV) was determined at shear rates of 10, 30, 45, 60, 120 and 150 s^−1^ at 37 °C, while plasma viscosity (PV) at 120 s^−1^ at 37 °C, with a cone-plate blood viscometer (LBY-N6, Precil, Beijing, China). The measurement of hematocrit was performed on a Sysmex XN2000 haematology analyzer (Sysmex Corporation, Kobe, Japan). For erythrocyte aggregation index (EAI), the value was calculated according to the equation EAI = V_L_/V_H_, in which V_L_ referred to the value of WBV at low shear rates of 10 s^−1^ and V_H_ was the value of WBV at high shear rates of 150 s^−1^ [25]. All hemorheological experiments for model assessment were completed within 3 h after blood collection.

### 4.5. Sample Preparation

Both urine and plasma samples were naturally thawed at room temperature prior to UPLC-Q/TOF-MS analysis. Acetonitrile (600 µL) was added to plasma sample (200 µL), and vortex-mixed vigorously for 2 min. After settled at room temperature for 10 min, the mixture was centrifuged at 12,000 rpm for 10 min at 4 °C with a microcentrifuge (Thermo Fisher Scientific, Heraeus Fresco 21, Harz, Germany). Then, the supernatant (500 µL) was blow-dried under a gentle nitrogen purge at 37 °C. The dried plasma sample was dissolved in 100 µL of acetonitrile/water (4:1). For urine samples, after centrifugation at 12,000 rpm for 10 min at 4 °C, the supernatant (1 mL) was pipetted out for analysis. Meanwhile, 20 μL of the urine samples were mixed to obtain quality control (QC) sample for method validation. Moreover, 15 μL of the plasma samples were mixed to obtain QC sample for method validation. All the QCs were collected in a continuous mode for correction of the system. Advanced one-needle QC sample before injection, one needle QC for every five samples in the sample [26].

### 4.6. UPLC-Q/TOF-MS Conditions

The UPLC analysis was performed on a Waters ACQUITY UPLC System coupled with a Waters Xevo G2-S QTof Mass Spectrometer (Waters Co., Milford, MA, USA). An ACQUITY UPLC BEH C18 column (2.1 × 100 mm, 1.7 μm) from Waters Corporation (Waters Corporation, Wexford, Ireland) was used to carry on the chromatographic separation at 30 °C. The mobile phases consisted of eluent A (0.1% formic acid in water, *v*/*v*) and eluent B (0.1% formic acid in acetonitrile, *v*/*v*) with flow rate of 0.4 mL/min with a liner gradient program: 10% B from 0 to 2 min, 10–90% B from 2 to 26 min, 90% B from 26 to 28 min, 90–10% B from 28 to 28.1 min. The autosampler was maintained at 4 °C. Mixtures of 10/90 and 90/10 water/acetonitrile were used as the strong wash and the weak wash solvent respectively. Every 3 µL sample solution was injected for each run.

Mass spectrometry was performed on a Xevo G2-S QToF instrument. The scan range was from 100–1200 Da. The optimized instrumental parameters were as follows: the capillary voltage floating at 2.6 kV (ESI^+^) or 2.2 kV (ESI^−^), cone voltage at 40 V for both ESI^+^ and ESI^−^ modes. The desolvation gas was set to 800 L/h at a temperature of 300 °C, the cone gas was set to 50 L/h and the source temperature was set to 120 °C. The mass spectrometry was operated in MS^E^ mode with the low energy of 6 V and the high energy of 20–40 V linearly. All analyses were acquired using the Lockspray to ensure accuracy and reproducibility. Leucine-enkephalin was used as the lockmass at a concentration of 300 ng/mL and flow rate of 20 μL/min. Data were collected in continuum mode, the lockspray interval was set at 10 s. QC sample ran several times during the analytical run to validate the system consistency [26]. The data acquisition rate was set to 1.0 s. All the acquisition of data was controlled by Waters Masslynx v4.1 software (Waters, Manchester, UK).

### 4.7. Data Analysis

For hemorheological investigation, data from model assessment were all ex-pressed as mean values ± standard deviation (SD), and multiple comparisons among groups were performed by one-way analyses of variance (ANOVA) by using the GraphPad 6.0 software (GraphPad Software Inc., San Diego, CA, USA). *P* < 0.05 was considered statistically significant.

For metabolomics analysis, the raw data were processed by the MarkerLynx XS V4.1 software for alignment, deconvolution, data reduction, normalization, etc, prior to principle component analysis (PCA) and orthogonal projections to latent structures discriminant analysis (OPLS-DA) [27]. As a result, the list of mass and retention time pairs with corresponding intensities for all the detected peaks from each data file. The main parameters were as follows: retention time range 0–28 min, mass range 100–1200 Da, mass tolerance 0.10, minimum intensity 5%, marker intensity threshold 2000 counts, mass window 0.10, retention time window 0.20, and noise elimination level 6. The resulting data were analyzed by PCA and OPLS-DA. Two parameters, R^2^Y and Q^2^ were used to evaluate the model. S-plots and VIP-plots were obtained via OPLS-DA analysis to find potential markers that significantly contributed to the difference among the groups. Biochemical databases, KEGG (http://www.kegg.com/), METLIN (http://metlin. scripps.edu/), HMDB (http://www.hmdb.ca/) and Metabo-Analyst (http://www.metaboanalyst.ca/), were used to identify potential markers. According to the data of MetaboAnalyst, the impact-value threshold was set above 0.10 and therefore the most important potential metabolic pathways were filtered out [15].

## 5. Conclusions

In the current study, XXT significantly decreased hemorheological indexes, including WBV, PV, hematocrit, thrombocytocrit and EAI in BSS rat, which indicated that pre-administration of XXT could significantly ameliorate BSS in vivo. Actually, XXT also exhibited excellent ability to regulate steroid hormone biosynthesis, arachidonic acid metabolism and sphingolipid metabolism. UPLC-Q/TOF-MS-based metabolomics could provide useful information for studying micromolecule endogenous metabolite changes in urine and plasma after one-week pretreatment with XXT in BSS rats. Fifteen potential markers were identified to be involved in arachidonic acid metabolism, steroid hormone biosynthesis and sphingolipid metabolism. These potential markers and their corresponding pathways further the studies on action mechanism of XXT in treating BSS.

## Figures and Tables

**Table 1:** Effects of XXT on whole blood and plasma viscosity in rats with BBS.

Group	Whole Blood Viscosity (mPa·s)						Plasma Viscosity (mPa·s) 120 s^−1^
	10 s^−1^	30 s^−1^	45 s^−1^	60 s^−1^	120 s^−1^	150 s^−1^	
NC	12.02 ± 1.39 **	8.26 ± 1.23 **	7.85 ± 0.65 **	7.05 ± 1.00 **	6.07 ± 0.74 **	5.78 ± 0.44 **	1.10 ± 0.18 **
BM	16.30 ± 2.12	10.04 ± 1.41	8.91 ± 0.87	8.21 ± 0.65	7.01 ± 0.66	6.74 ± 0.54	1.33 ± 0.08
XT	12.50 ± 1.79 *	8.84 ± 0.93 *	8.00 ± 0.89 *	7.51 ± 0.64 *	6.37 ± 0.63 *	6.04 ± 0.67 *	1.24 ± 0.80 *

* *P* < 0.05, ** *P* < 0.01 versus BM group. NC represents normal control group; BM represents BSS model group; XT represents XXT treated group.

**Table 2:** Effects of XXT on hematocrit and EAI in rats with BSS.

Group	Hematocrit (%)	EAI
NC	0.35 ± 0.05 **	2.09 ± 0.25 *
BM	0.45 ± 0.10	2.41 ± 0.12
XT	0.37 ± 0.07 *	2.09 ± 0.35 *

* *P* < 0.05, ** *P* < 0.01 versus BM group. NC represents normal control group; BM represents BSS model group; XT represents XXT treated group.

**Table 3:** Identification results of potential markers in urine and plasma.

No.	RT (min)	Measured Mass (Da)	VIP	Formula	Error (ppm)	Identification	HMDB ID	Pathway	Content Level	Mode
1	7.60	333.2421	2.83	C_21_H_32_O_3_	0.6	7α-Hydroxypregnenolone	HMDB60424	Steroid hormone biosynthesis	BM > XT ≈ NC	urine-ESI^+^
2 *	8.17	365.2344	3.18	C_20_H_32_O_3_	5.5	5-HETE	HMDB11134	Arachidonic acid metabolism	BM > XT ≈ NC	urine-ESI^−^
3	8.91	331.2271	5.98	C_21_H_30_O_3_	3.0	17-Hydroxyprogesterone	HMDB00374	Steroid hormone biosynthesis	BM > XT ≈ NC	urine-ESI^+^
4 *	8.91	365.2340	4.39	C_20_H_32_O_3_	1.4	16(*R*)-HETE	HMDB04680	Arachidonic acid metabolism	BM > XT ≈ NC	urine-ESI^−^
5	9.59	287.2018	2.87	C_19_H_26_O_2_	2.8	Androstenedione	HMDB00053	Steroid hormone biosynthesis	BM > XT ≈ NC	urine-ESI^+^
6 *	9.81	347.2221	8.13	C_21_H_30_O_4_	1.7	21-Deoxycortisol	HMDB04030	Steroid hormone biosynthesis	BM > XT ≈ NC	urine-ESI^+^
7	9.82	363.2180	1.33	C_20_H_30_O_3_	3.0	Leukotriene A4	HMDB01337	Arachidonic acid metabolism	BM > XT ≈ NC	urine-ESI^−^
8 *	9.91	349.2375	10.27	C_21_H_32_O_4_	1.1	3α,21-Dihydroxy-5β-pregnane-11,20-dione	HMDB06755	Steroid hormone biosynthesis	BM > XT ≈ NC	urine-ESI^+^
9 *	9.94	365.2336	5.12	C_20_H_32_O_3_	8.8	19(*S*)-HETE	HMDB11136	Arachidonic acid metabolism	BM > XT ≈ NC	urine-ESI^−^
10 *	11.41	331.2269	3.87	C_21_H_30_O_3_	6.9	Deoxycorticosterone	HMDB00016	Steroid hormone biosynthesis	BM > XT ≈ NC	urine-ESI^+^
11	12.64	333.2417	2.66	C_21_H_32_O_3_	0.3	21-Hydroxypregnenolone	HMDB04026	Steroid hormone biosynthesis	BM > XT ≈ NC	urine-ESI^+^
12 *	12.82	347.2235	11.94	C_21_H_30_O_4_	6.3	Cortexolone	HMDB00015	Steroid hormone biosynthesis	BM > XT ≈ NC	urine-ESI^+^
13 *	13.19	318.3018	1.72	C_18_H_39_NO_3_	4.4	Phytosphingosine	HMDB04610	Sphingolipid metabolism	BM < XT ≈ NC	urine-ESI^+^
14 *	15.62	302.3068	2.58	C_18_H_39_NO_2_	5.3	Sphinganine	HMDB00269	Sphingolipid metabolism	BM < XT ≈ NC	urine-ESI^+^
15 *	22.68	305.2490	1.12	C_20_H_32_O_2_	2.0	Arachidonic acid	HMDB01043	Arachidonic acid metabolism	BM > XT ≈NC	urine-ESI^+^

* Compared with standards.

## Supplementary Materials

The following are available online. PART I: MS/MS spectra of standards and identified potential markers. PART II: MS/MS spectra of the identified potential markers by comparing accurate molecular weight and tandem mass spectrometry with HMDB database.
