# Supplementary material for: Comprehensive Metabolomics Analysis of Xueshuan Xinmaining Tablet in Blood Stasis Model Rats Using UPLC-Q/TOF-MS
Source: Molecules. 2018 Jul 6;23(7):1650. doi: 10.3390/molecules23071650 (PMC6099806; doi:10.3390/molecules23071650)
Supplement: Supplementary file 1 [file molecules-23-01650-s001.pdf]

Supplementary Material

PART I: MS/MS spectra of *standards* and *identified potential markers*

There were ten of the putative markers were confirmed with available reference standards by matching their retention time and accurate mass measurement. The MS/MS spectra of each standard and corresponding identified potential marker are shown in Figure 1-10.

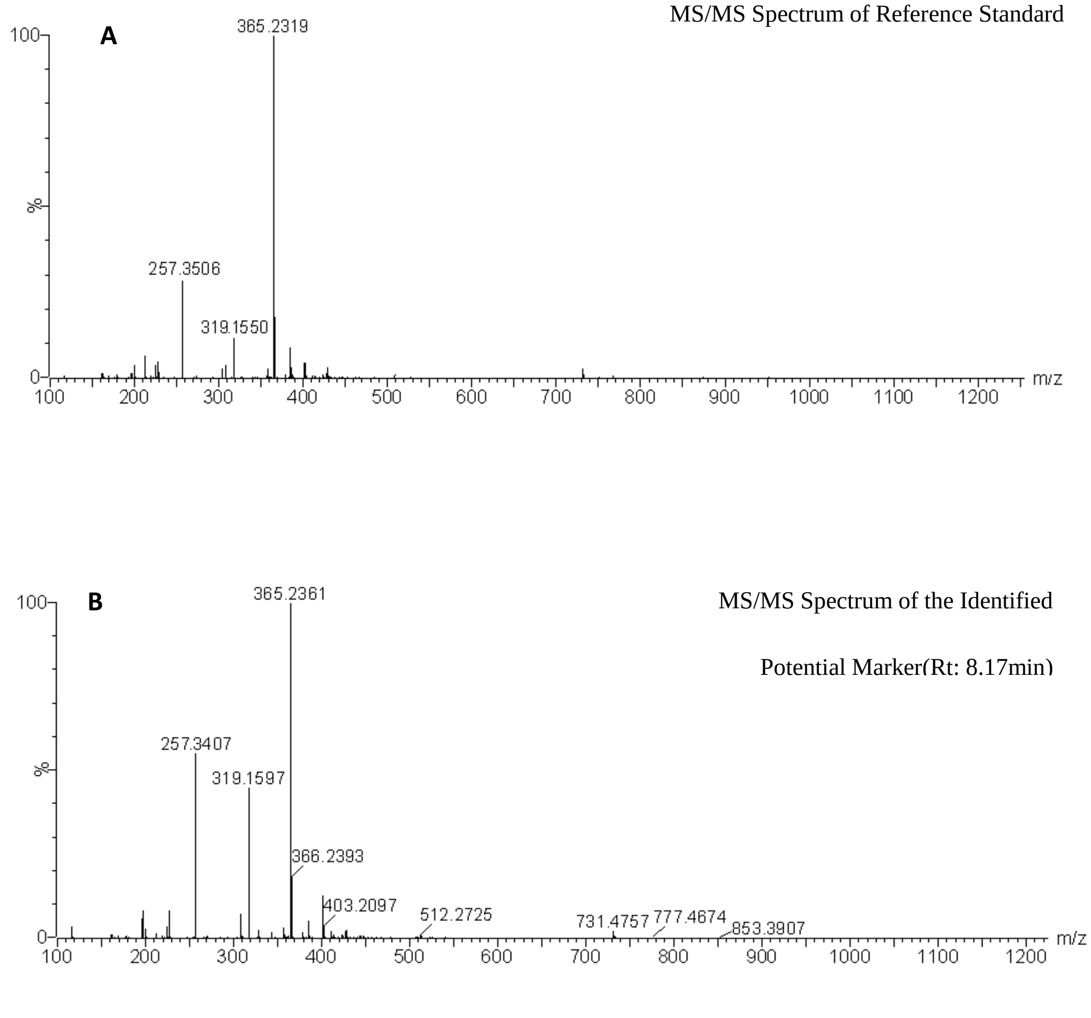

Figure S1. The MS/MS spectra of standard 5-Hete (A) and identified potential marker (B)

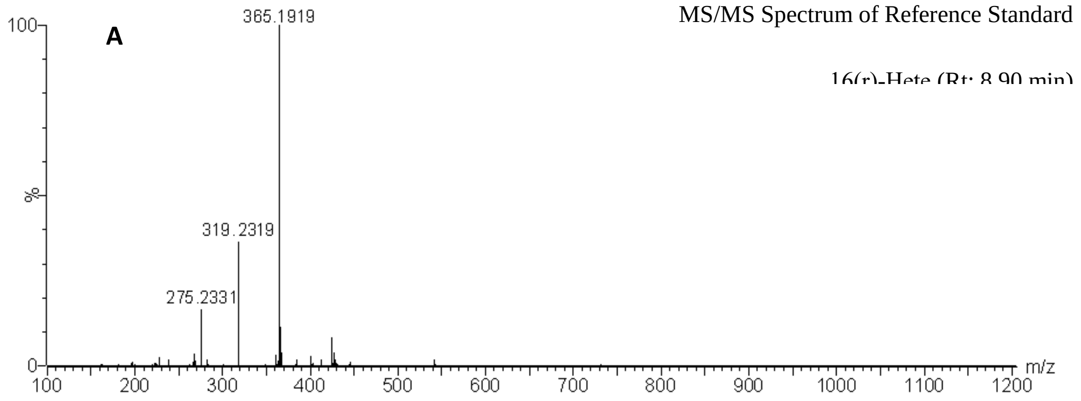

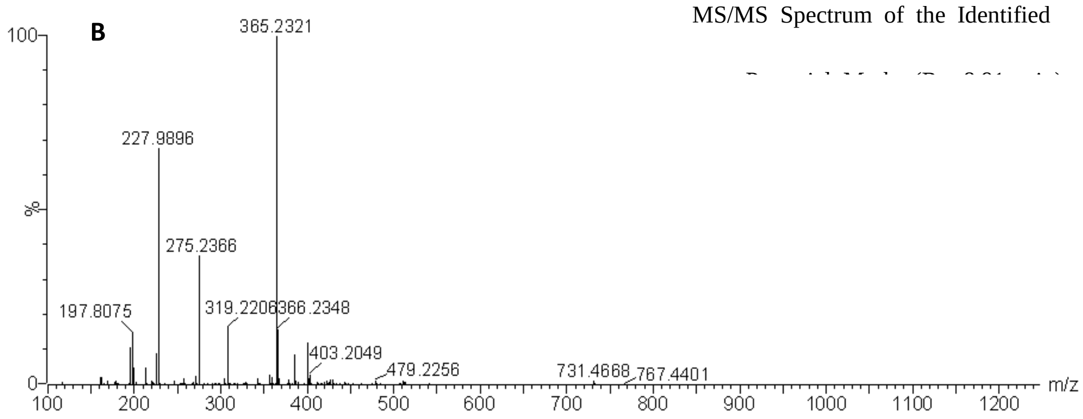

Figure S2. The MS/MS spectra of standard 16(r)-Hete (A) and identified potential marker (B)

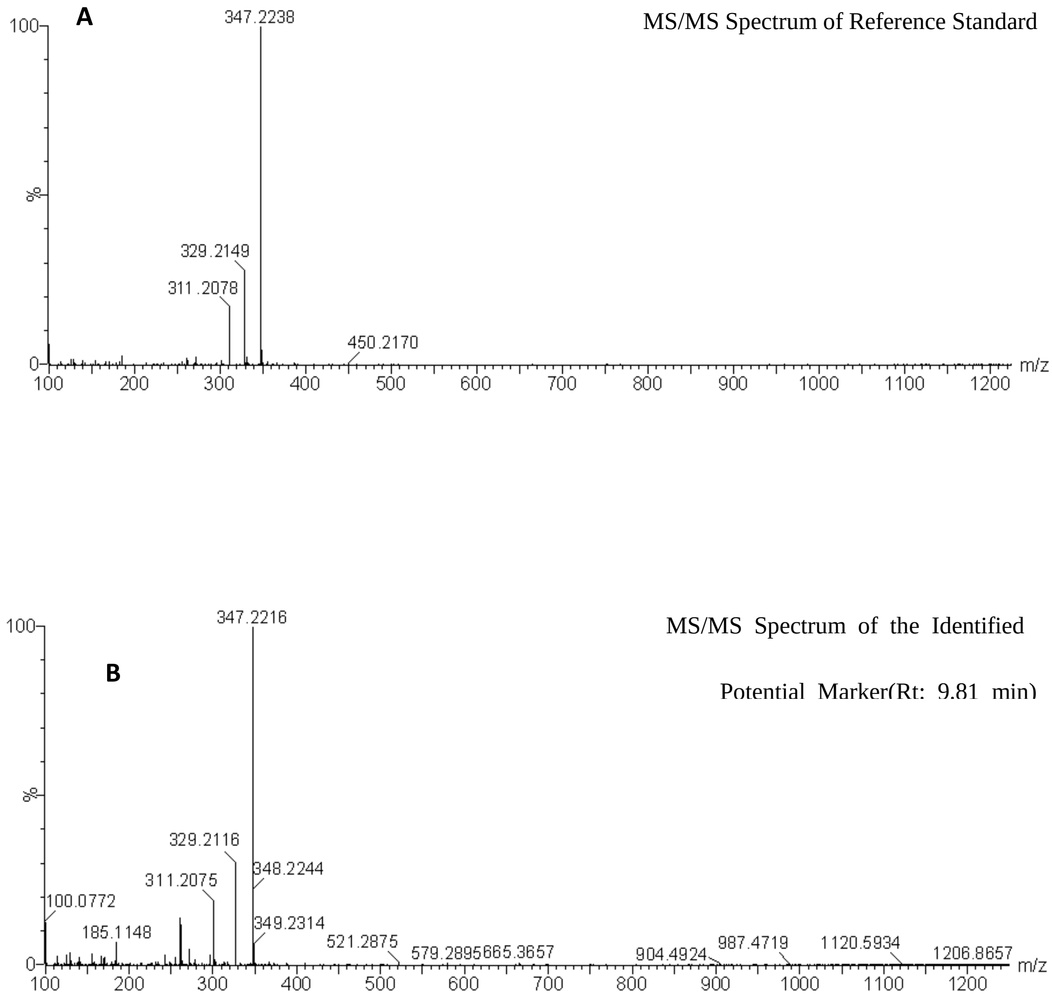

Figure S3. The MS/MS spectra of standard 21-Deoxy-Cortisol (A) and identified potential marker (B)

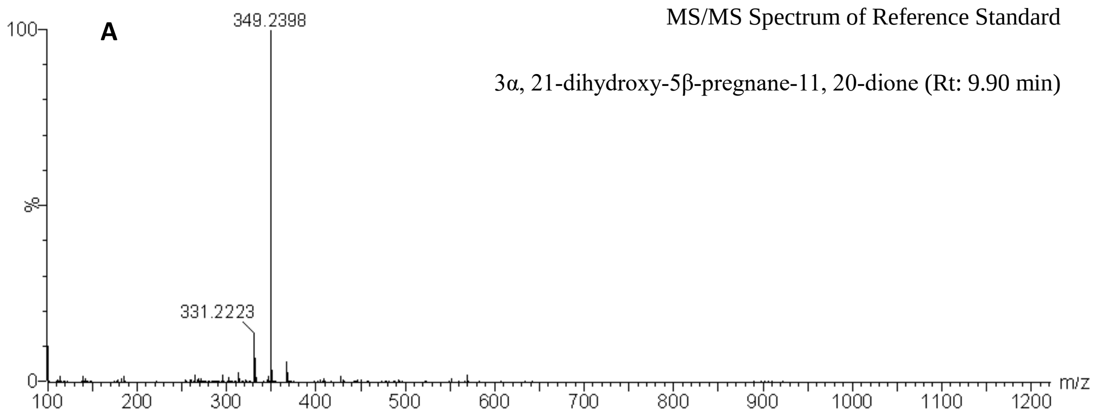

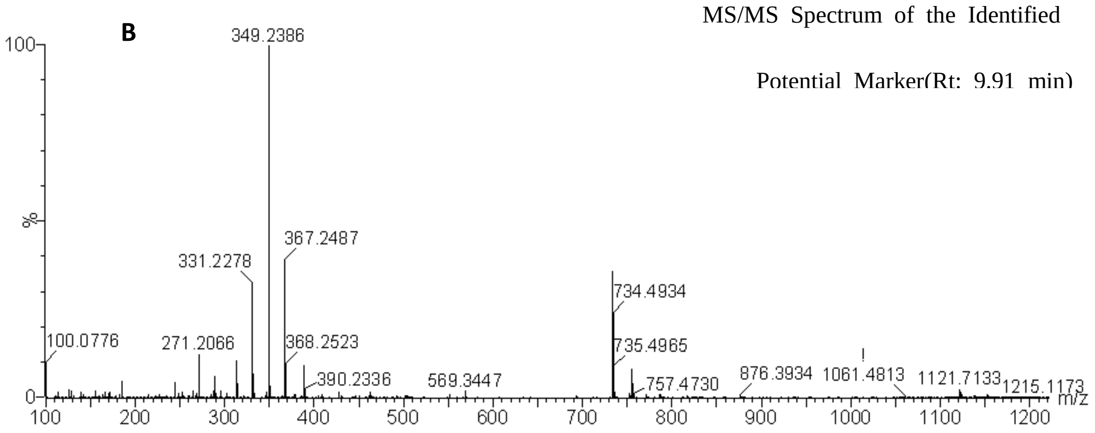

Figure S4. The MS/MS spectra of standard 3α, 21-dihydroxy-5β-pregnane-11, 20-dione (A) and identified potential marker (B)

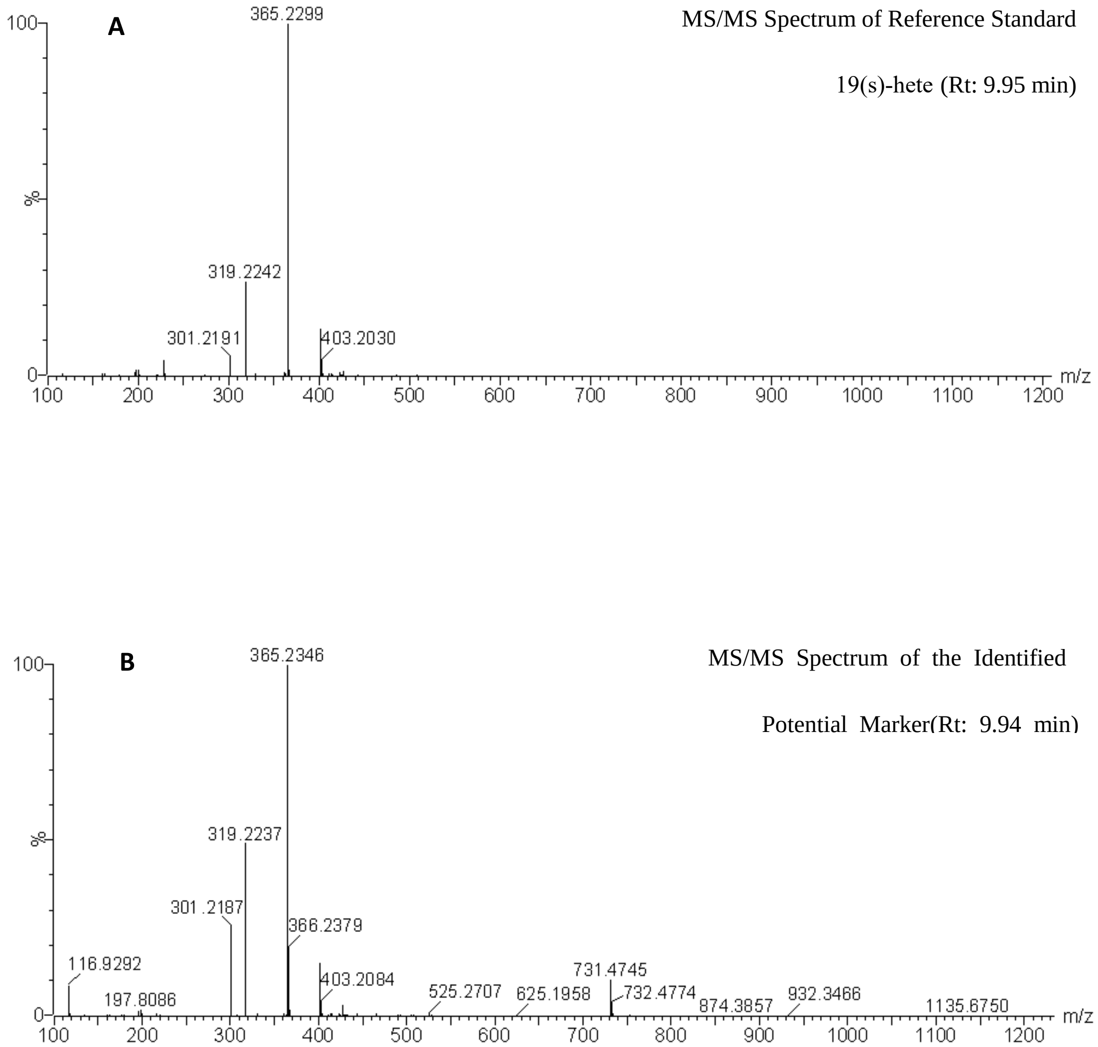

Figure S5. The MS/MS spectra of standard 19(s)-hete (A) and Identified potential marker (B)

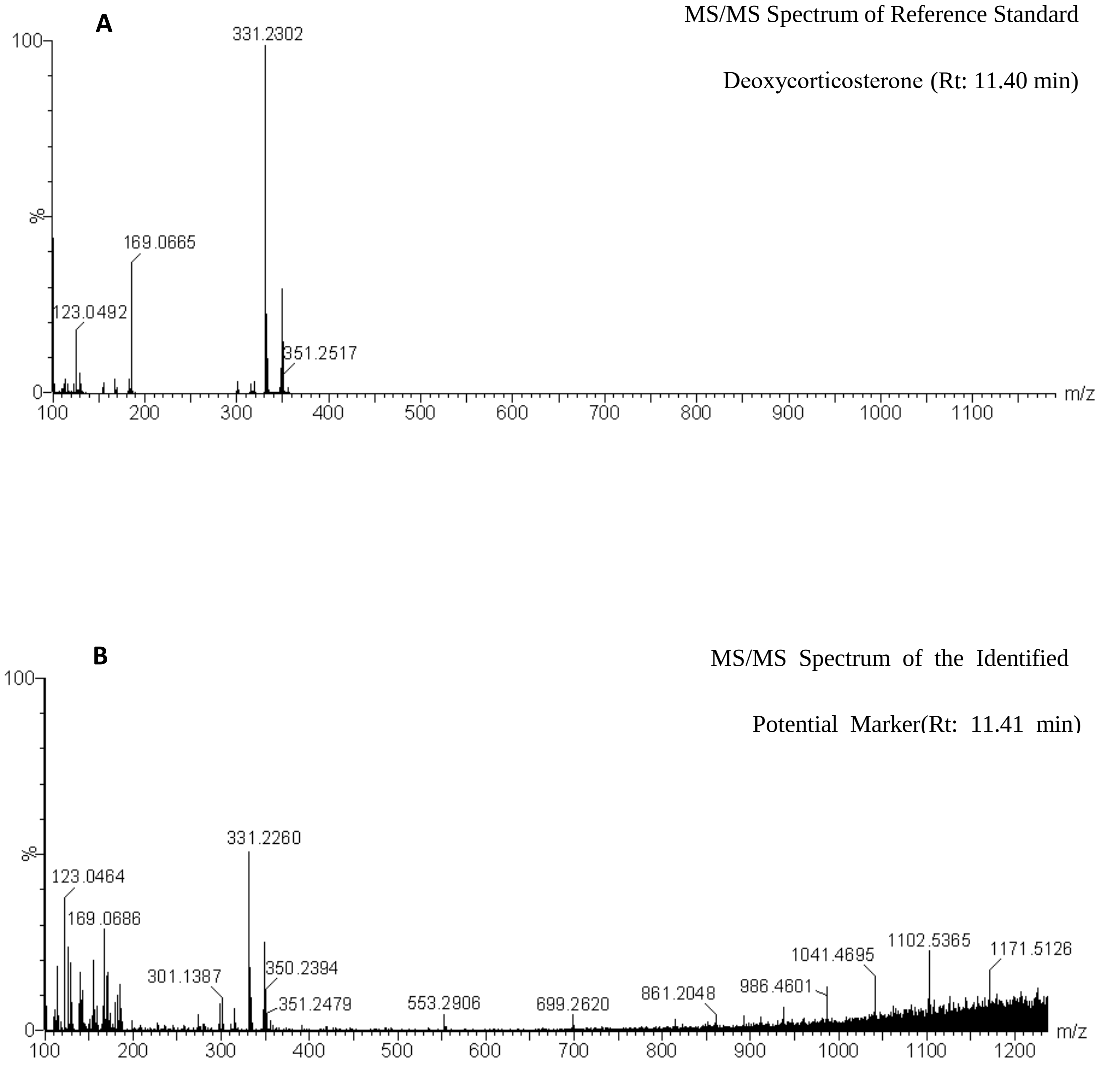

Figure S6. The MS/MS spectra of standard Deoxycorticosterone (A) and identified potential marker (B)

A

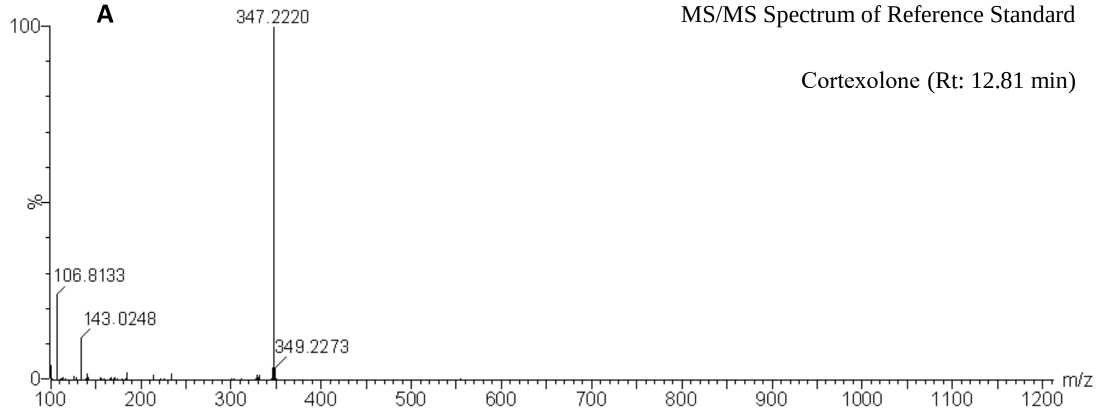

B

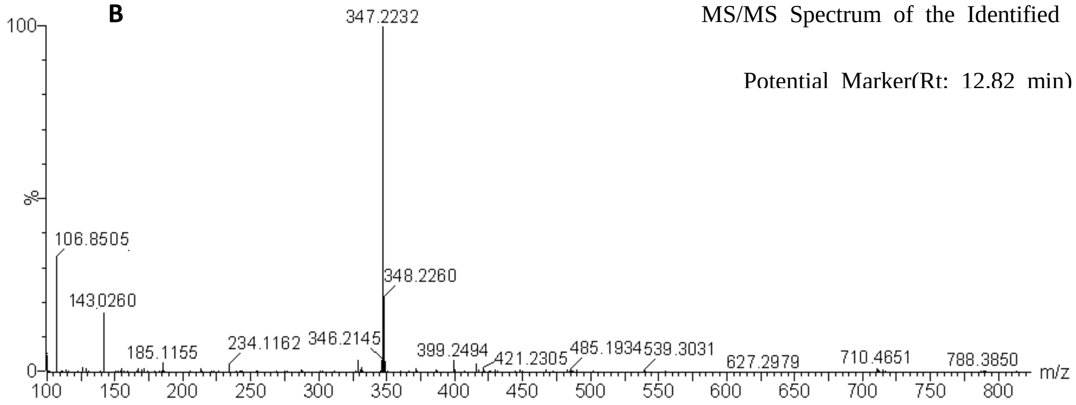

Figure S7. The MS/MS spectra of standard Cortexolone (A) and identified potential marker (B)

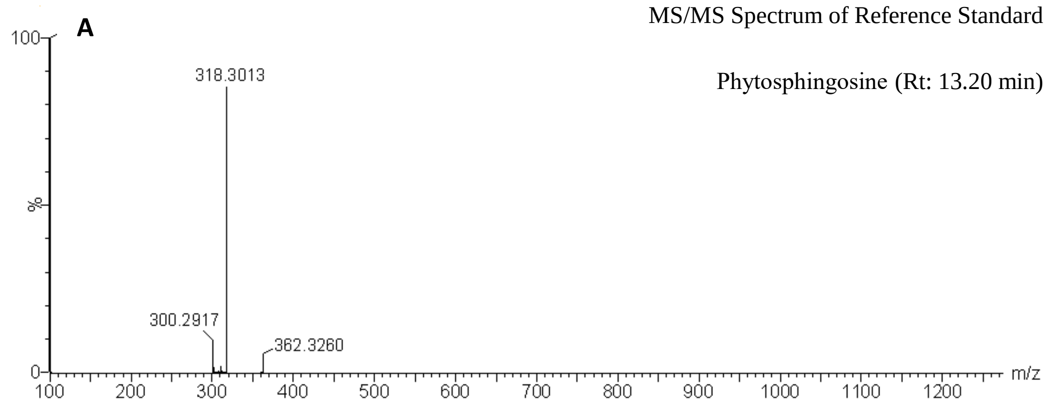

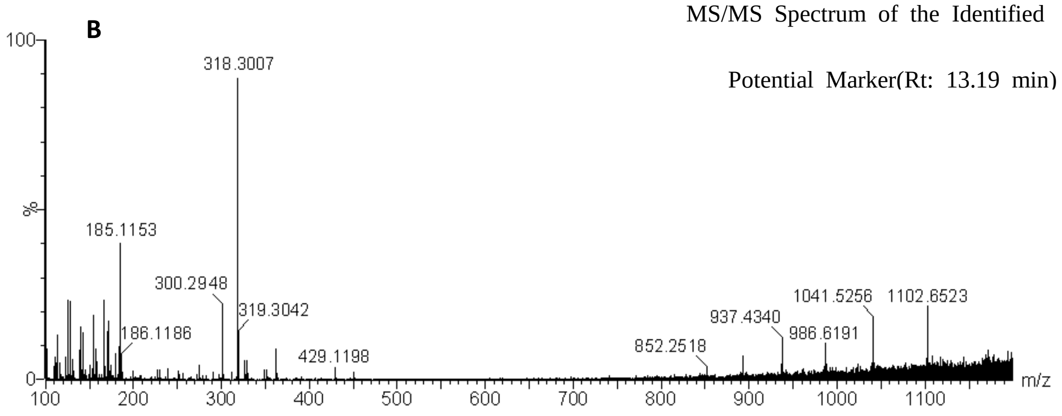

Figure S8. The MS/MS spectra of standard Phytosphingosine (A) and identified potential marker (B)

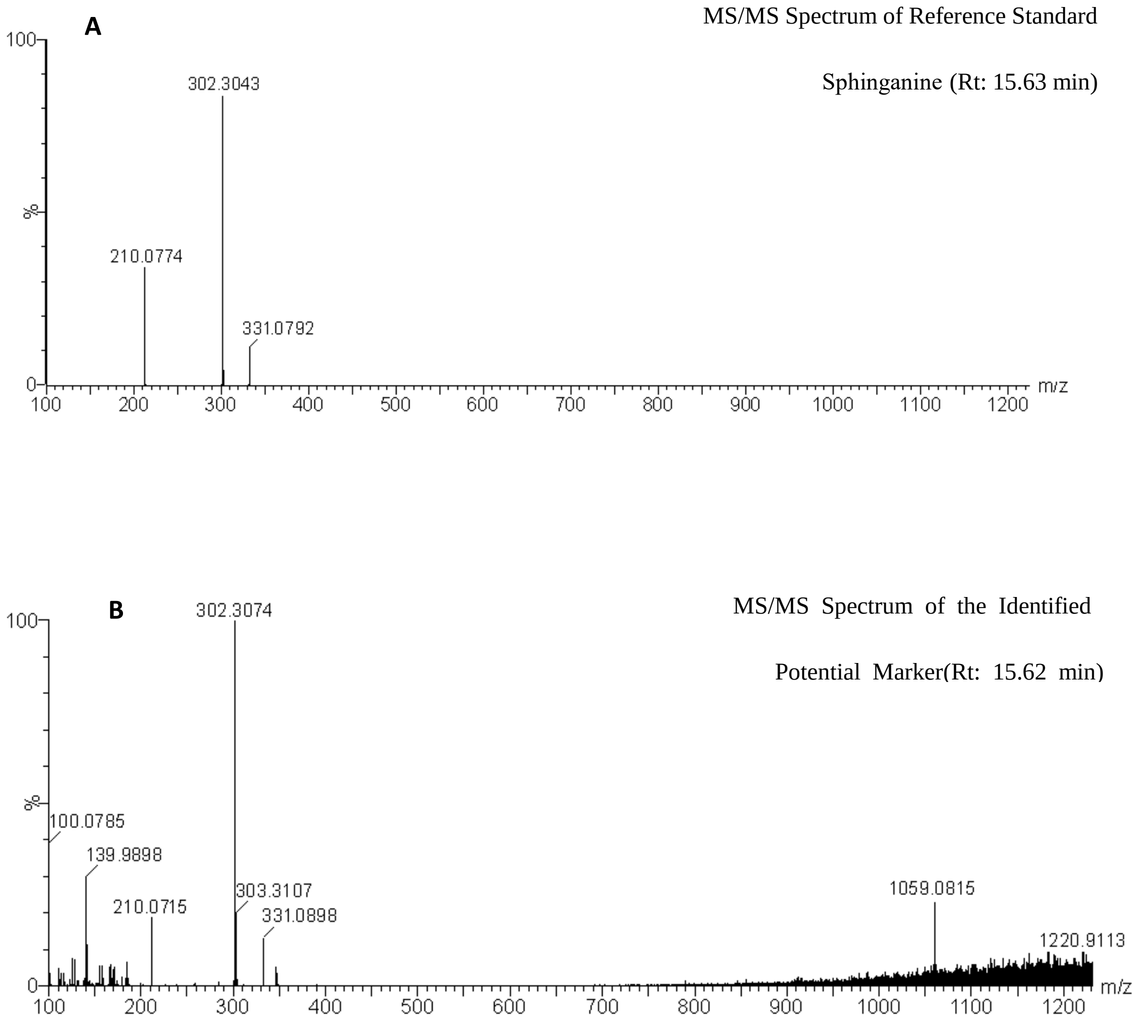

Figure S9. The MS/MS spectra of standard Sphinganine (A) and.

identified potential marker (B)

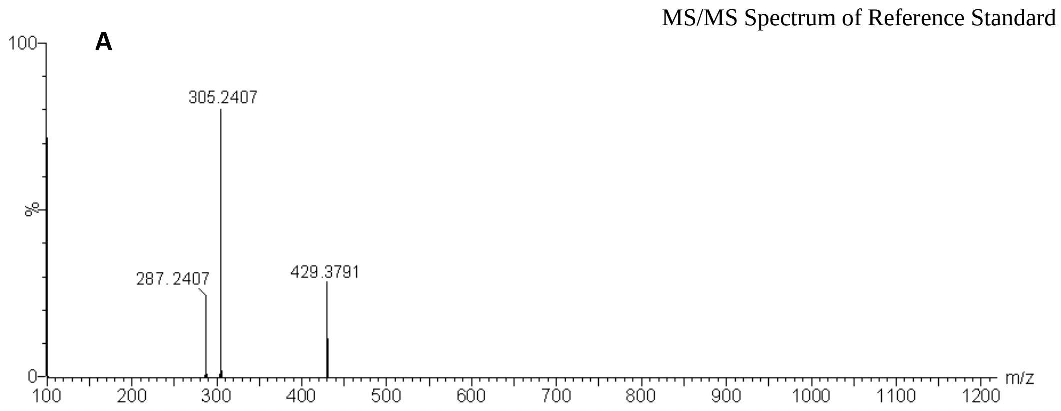

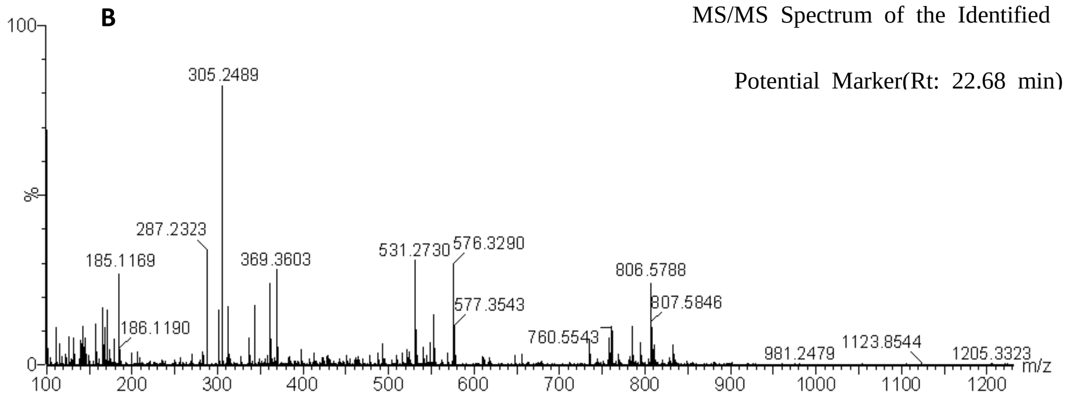

Figure S10. The MS/MS spectra of standard Arachidonic acid (A) and identified potential marker (B)

PART II: MS/MS spectra of the identified potential markers by comparing accurate molecular weight and tandem mass spectrometry with HMDB database

There were other five putative structures of the metabolites were identified by comparing accurate molecular weight and tandem mass spectrometry obtained in the study with the information recorded in biochemical database (HMDB).

The MS/MS spectra of each identified potential marker and corresponding information recorded in HMDB database are shown in Figure 11-15.

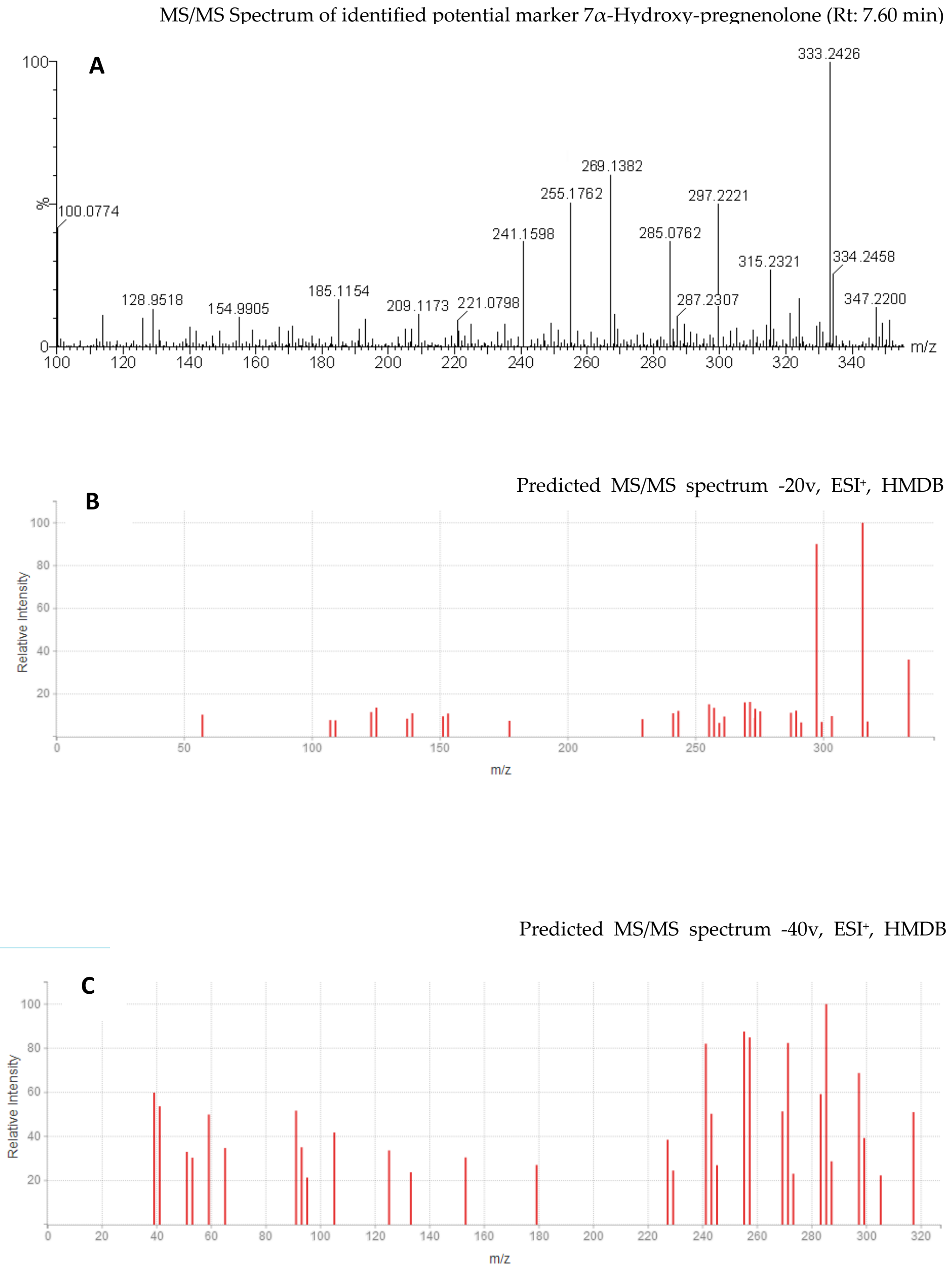

Figure S11. The MS/MS spectra of identified potential marker 7α-Hydroxy-pregnenolone (A) and the MS/MS spectrum in HMDB database (B, C)

MS/MS Spectrum of identified potential marker 17-Hydroxy-proesterone (Rt: 8.91 min)

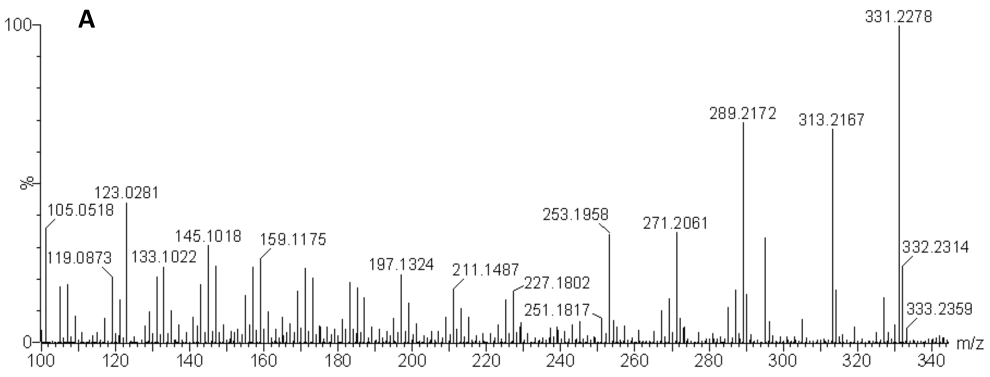

Predicted MS/MS spectrum -20v, ESI+, HMDB

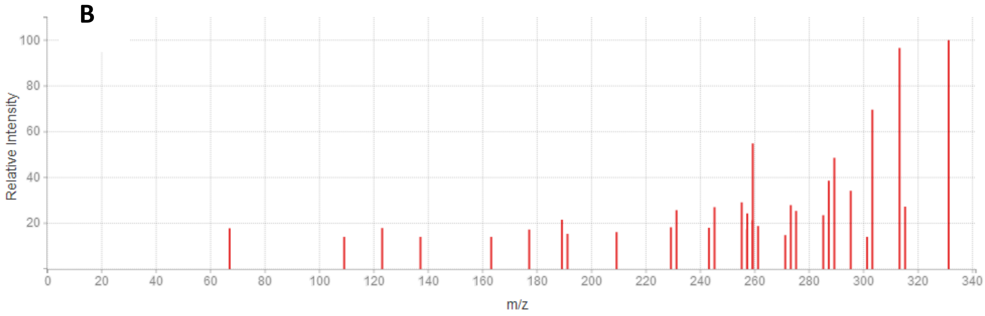

Predicted MS/MS spectrum -40v, ESI+, HMDB

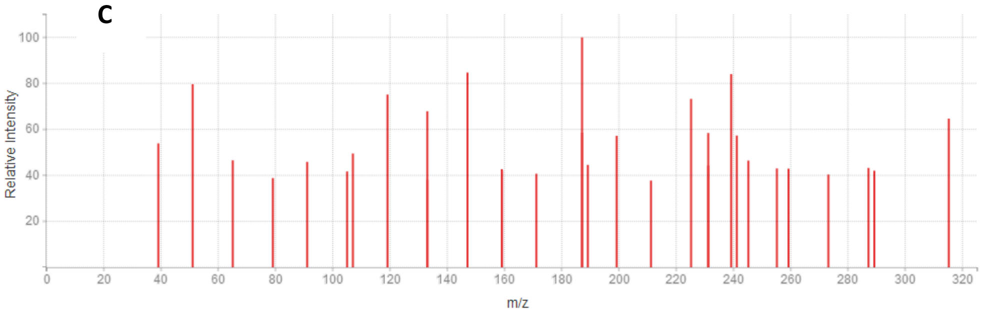

Figure S12. The MS/MS spectra of identified potential marker 17-Hydroxy-proesterone (A) and the MS/MS spectrum in HMDB database (B, C)

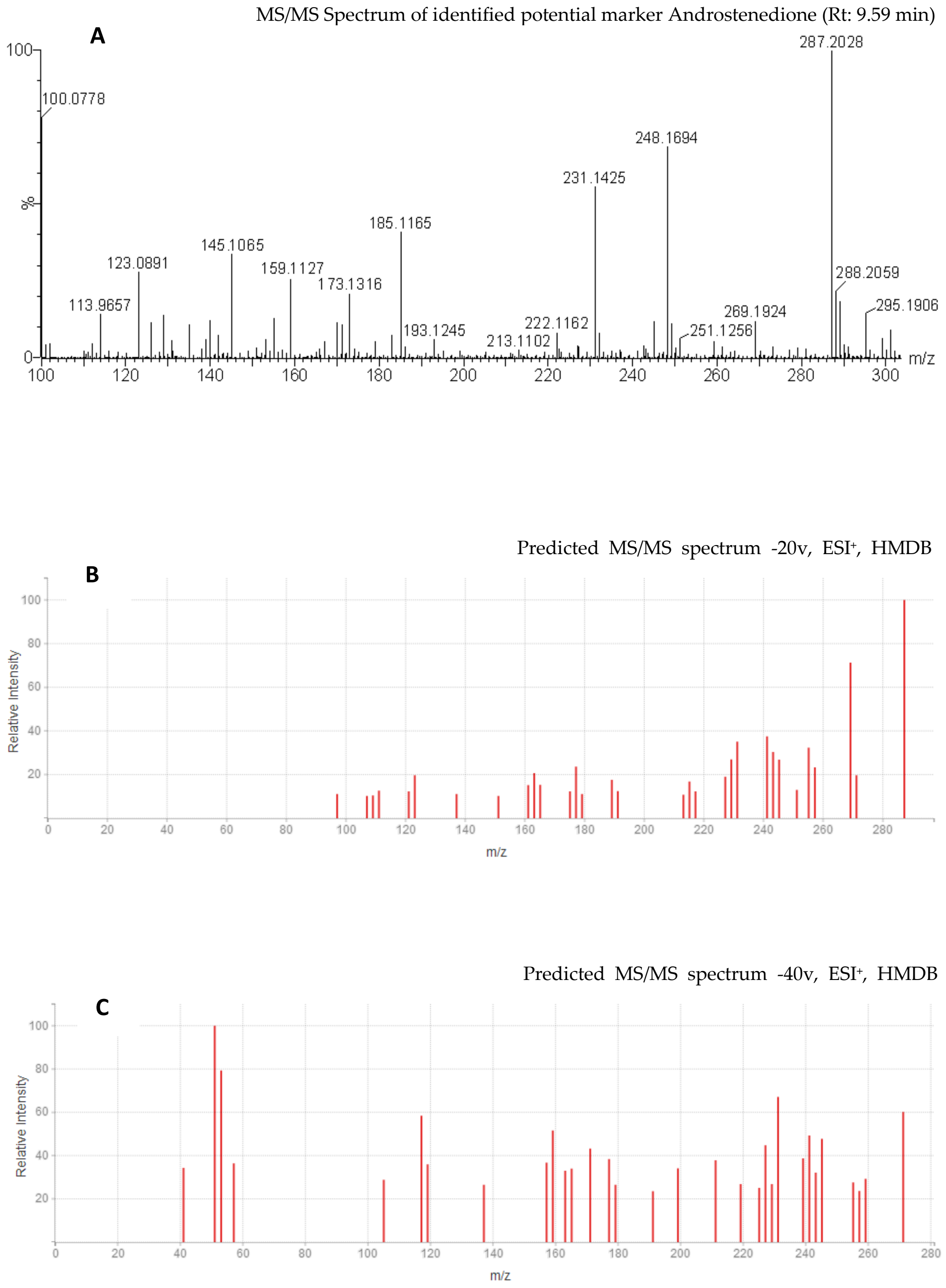

Figure S13. The MS/MS spectra of identified potential marker Androstenedione (A) and the MS/MS spectrum in HMDB database (B, C)

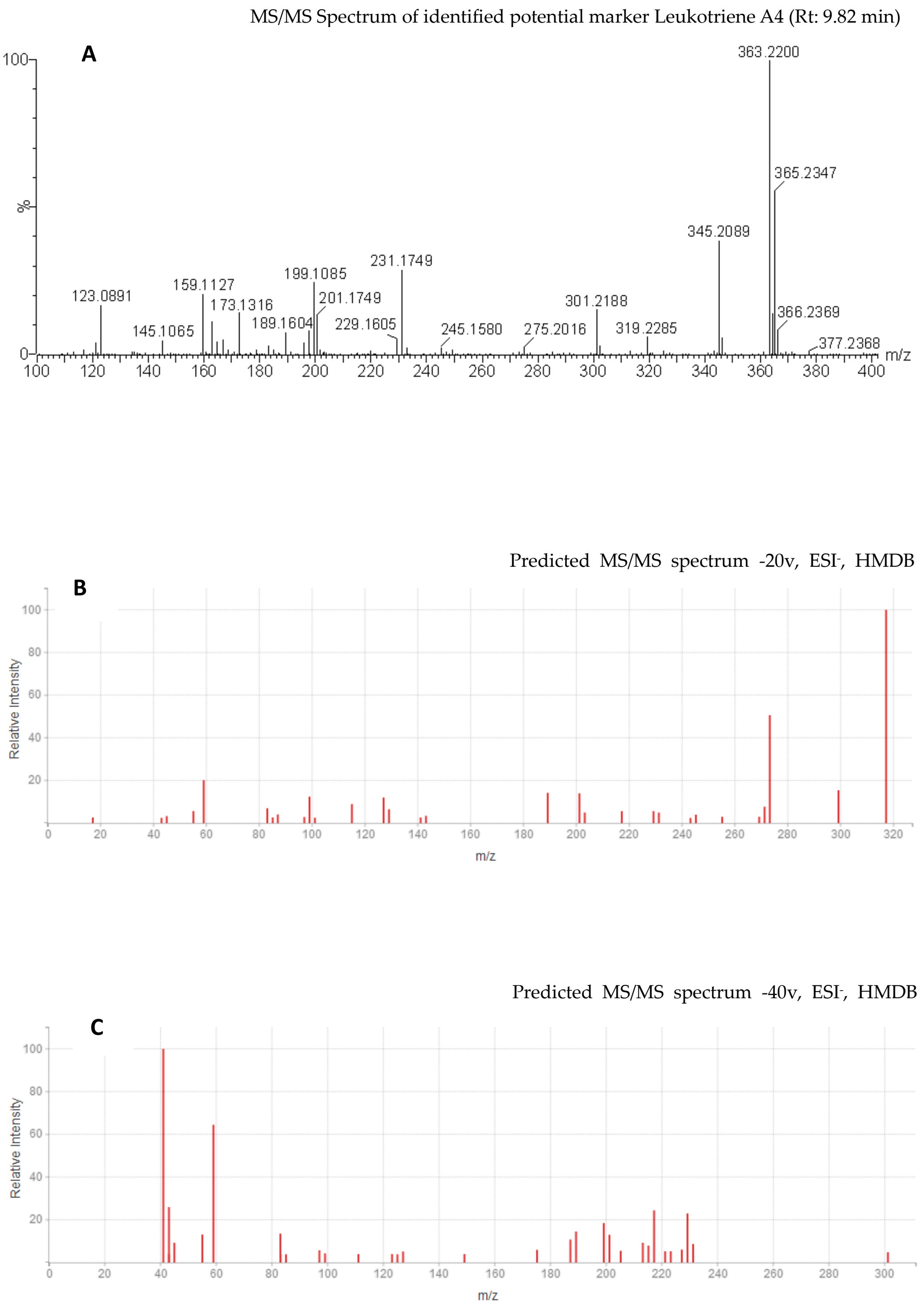

Figure S14. The MS/MS spectra of identified potential marker Leukotriene A4 (A) and the MS/MS spectrum in HMDB database (B, C)

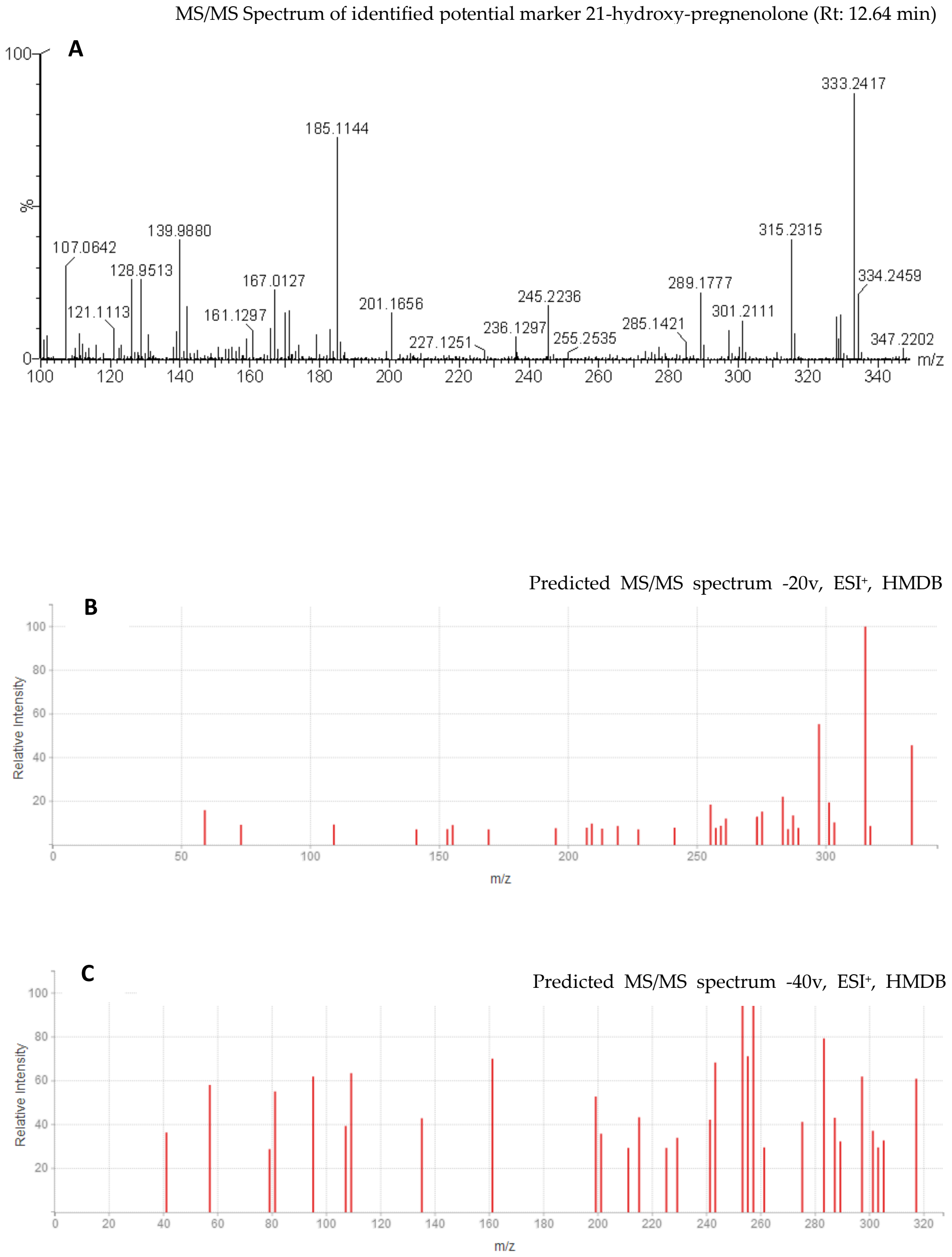

Figure S15. The MS/MS spectra of identified potential marker 21-hydroxy-pregnenolone (A) and the MS/MS spectrum in HMDB database (B, C)
